# Supplementary figures and images for: Inserting CTL Epitopes of the Viral Nucleoprotein to Improve Immunogenicity and Protective Efficacy of Recombinant Protein against Influenza A Virus
Source: Biology (Basel). 2024 Oct 7;13(10):801. doi: 10.3390/biology13100801 (PMC11505154; doi:10.3390/biology13100801)

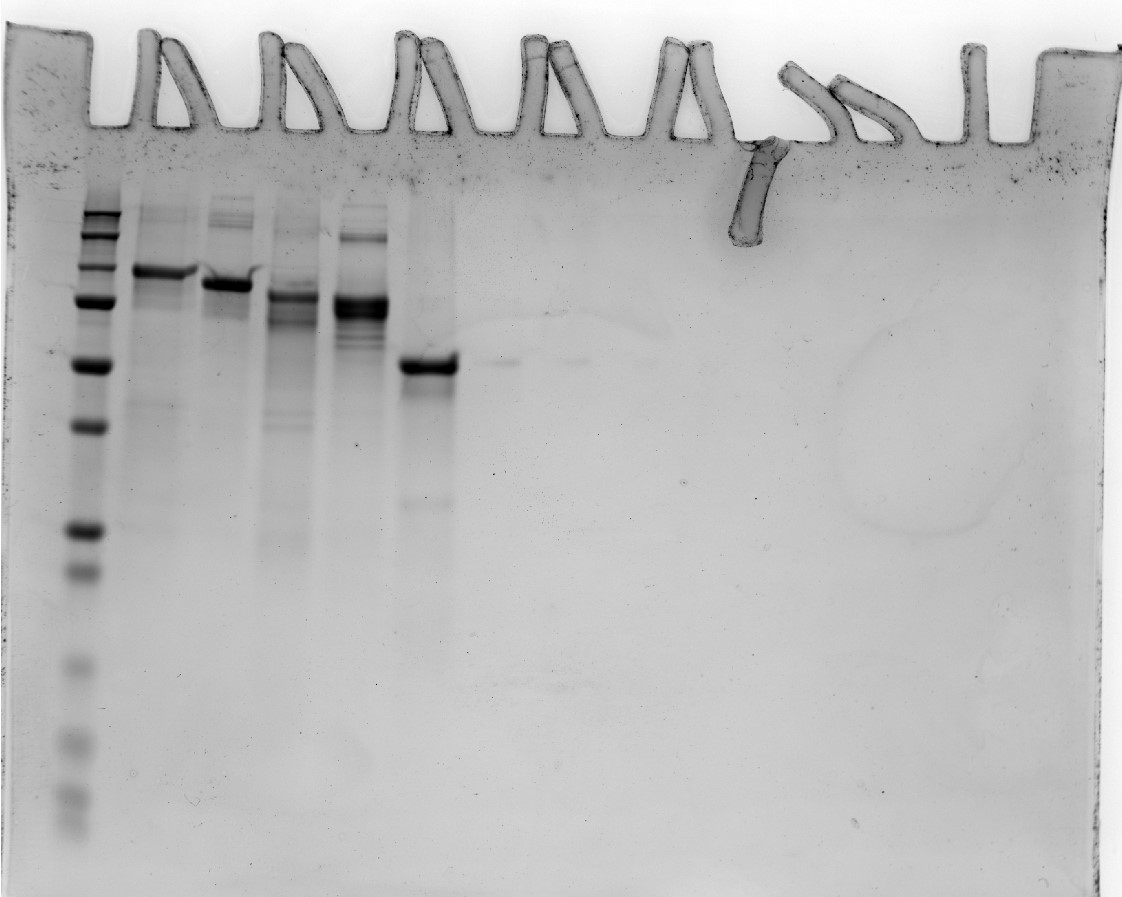

Supplement: Supplementary file 1 [file biology-13-00801-s001.zip › S1.jpg]

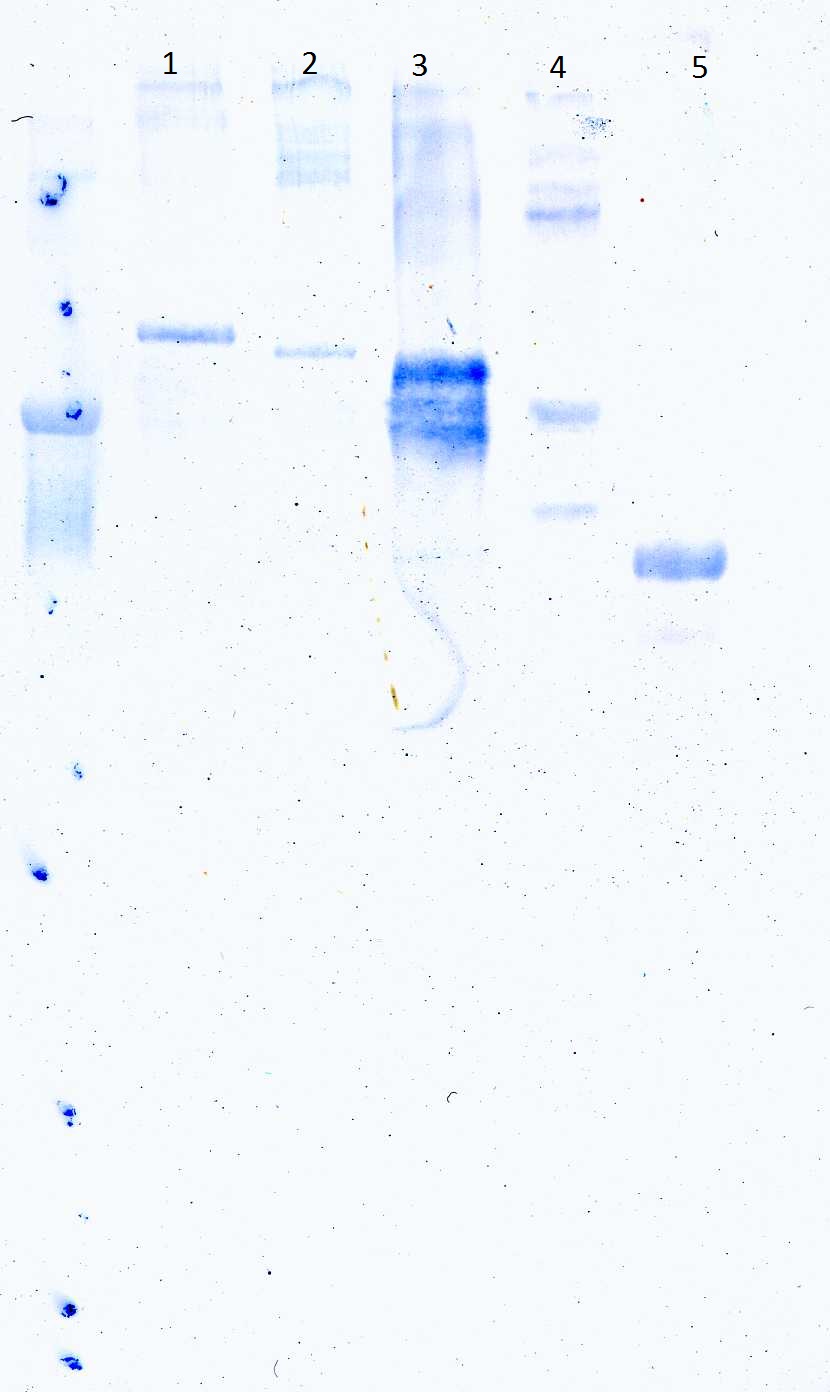

Supplement: Supplementary file 1 [file biology-13-00801-s001.zip › S2.jpg]

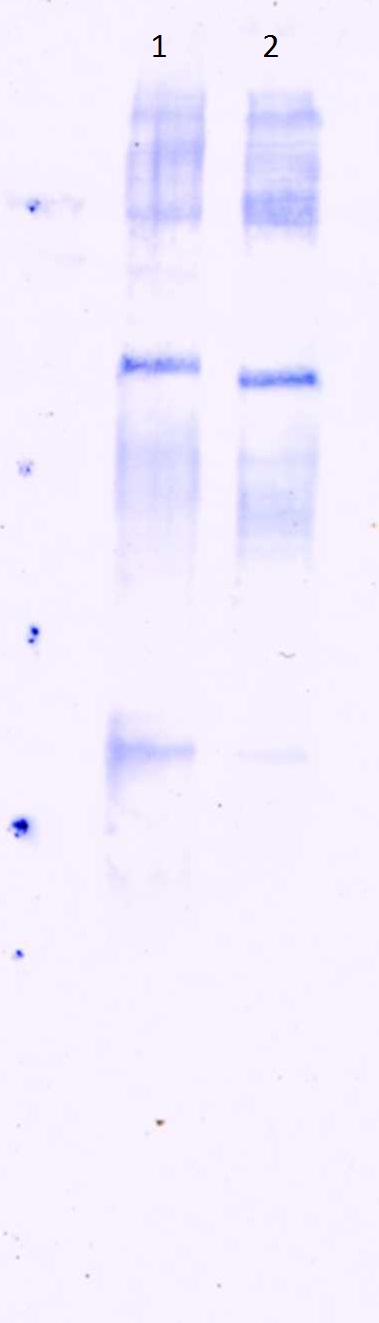

Supplement: Supplementary file 1 [file biology-13-00801-s001.zip › S3.jpg]

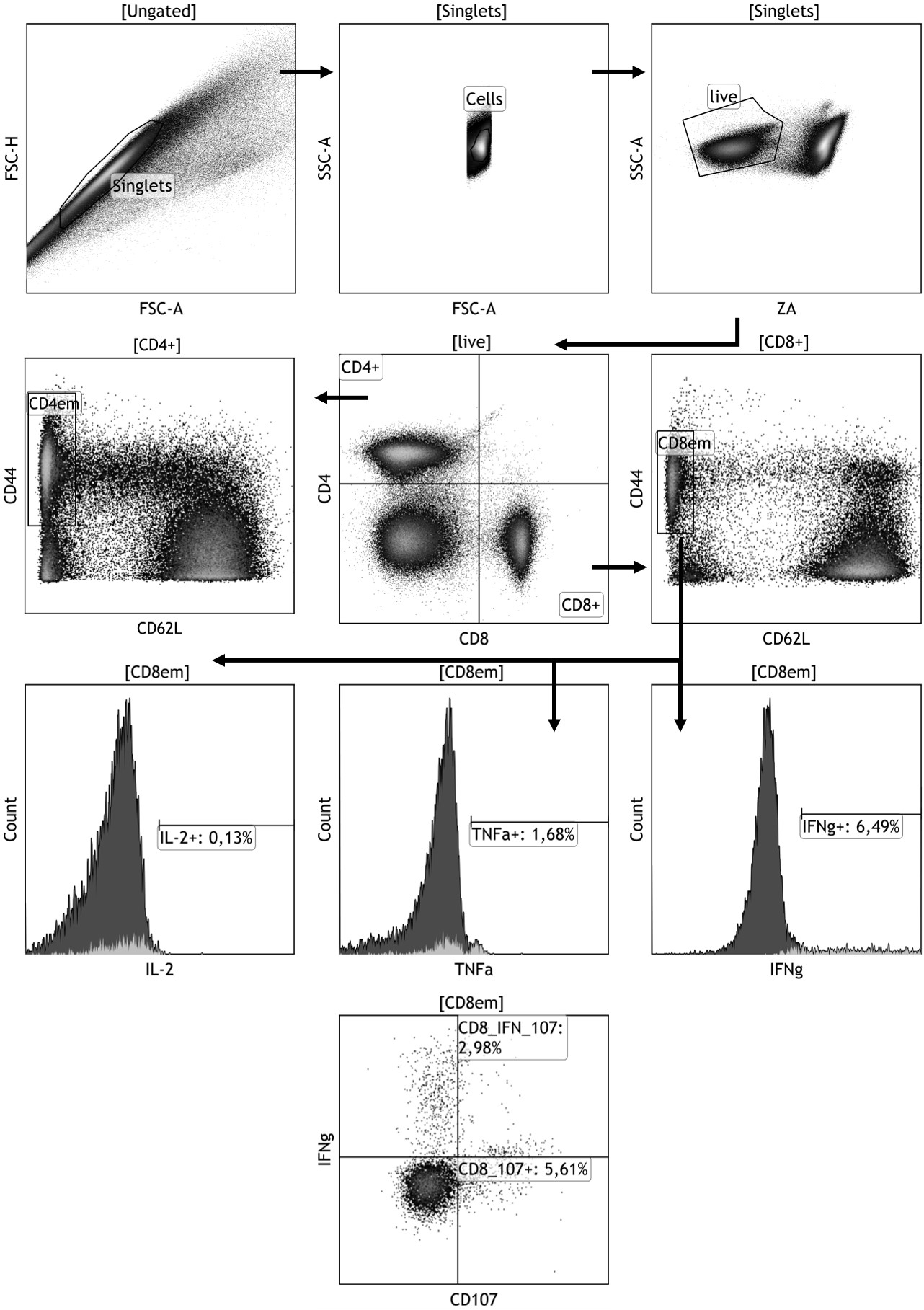

Supplement: Supplementary file 1 [file biology-13-00801-s001.zip › S4.tif]

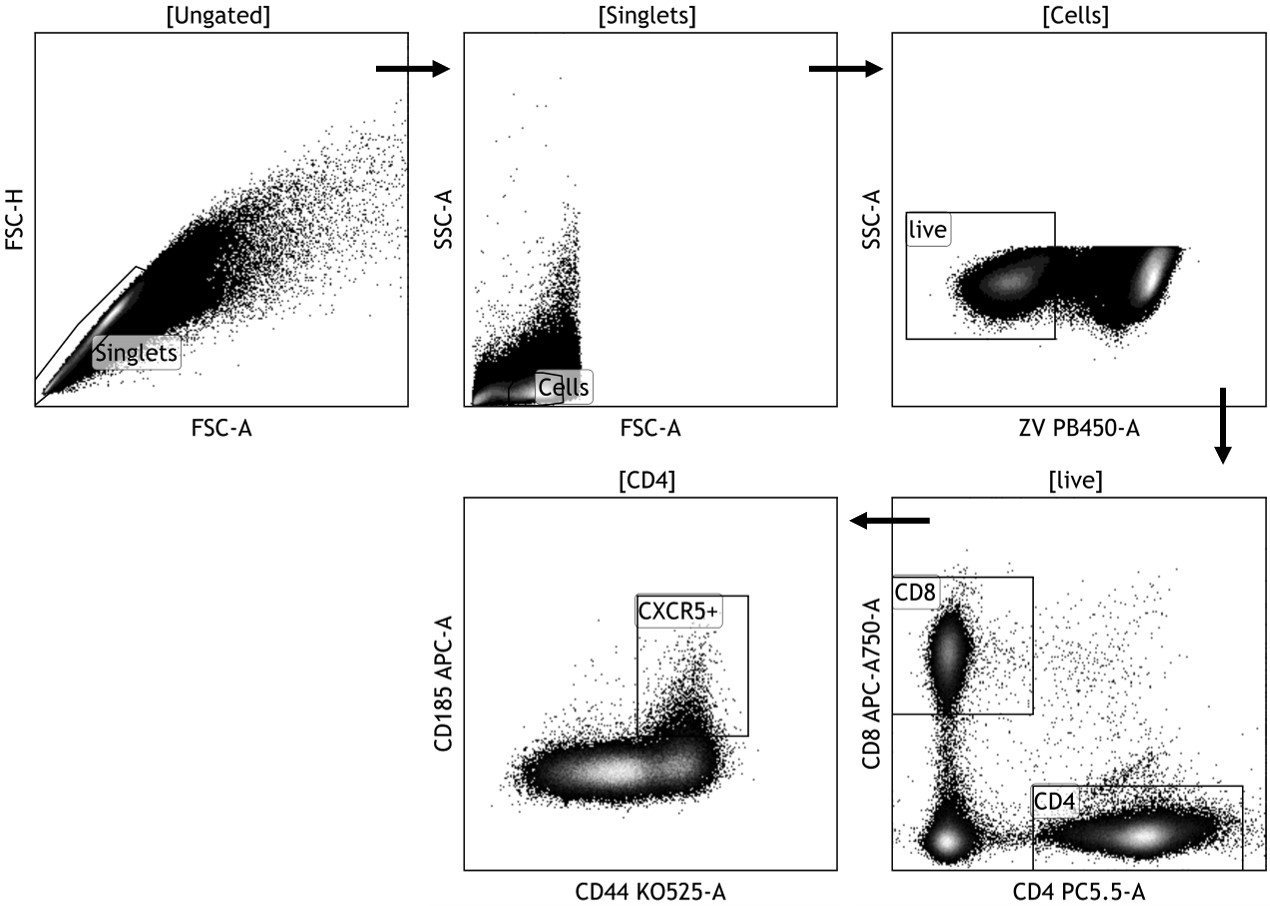

Supplement: Supplementary file 1 [file biology-13-00801-s001.zip › S5.tif]
